# Chemiluminescence Immunoassay and Enzyme-Linked Immunosorbent Assay in the Diagnosis of Pemphigoid and Pemphigus: A Comparative Study

**DOI:** 10.3390/ijms27146272

**Published:** 2026-07-14

**Authors:** Yan Wang, Zhe Fan, Jie Zhang, Min Bai, Jin-Li Qin, Chao-Jun Hu, Ya-Gang Zuo

**Affiliations:** 1 State Key Laboratory of Complex Severe and Rare Diseases, Department of Dermatology, Peking Union Medical College Hospital, Chinese Academy of Medical Sciences and Peking Union Medical College, Beijing 100730, China; wy1997edu@163.com (Y.W.); fanz20@mails.tsinghua.edu.cn (Z.F.); zhangj5250318@163.com (J.Z.); 2 Department of Rheumatology and Clinical Immunology, Peking Union Medical College Hospital, Chinese Academy of Medical Sciences and Peking Union Medical College, Beijing 100730, China; baimin1998@163.com (M.B.); 15313708761@163.com (J.-L.Q.)

**Keywords:** chemiluminescence immunoassay, enzyme-linked immunosorbent assay, bullous pemphigoid, pemphigus, autoantibody

## Abstract

Autoimmune bullous diseases (AIBDs), including pemphigus vulgaris (PV), pemphigus foliaceus (PF), and bullous pemphigoid (BP), are mediated by autoantibodies against desmogleins (DSG1, DSG3) or hemidesmosomal proteins (BP180, BP230). While enzyme-linked immunosorbent assay (ELISA) is commonly applied for antibody detection, chemiluminescent immunoassay (CLIA) offers advantages such as a broader dynamic range and higher automation. To compare the diagnostic performance of CLIA and ELISA for detecting autoantibodies in patients with AIBDs, we collected 255 serum samples (92 controls, 84 BP, 69 PV, 10 PF) and 85 blister fluid samples (42 controls, 43 BP). Serum was tested for anti-BP180, anti-BP230, anti-DSG1, and anti-DSG3 antibodies using both assays; blister fluid was tested for anti-BP180 and anti-BP230. Using established cut-offs, the assays demonstrated good diagnostic accuracy for anti-BP180, anti-DSG1, and anti-DSG3 in serum, and excellent concordance (>90%). Receiver operating characteristic analysis revealed acceptable to excellent diagnostic value for both sample types, with CLIA generally achieving a higher area under the curve. Spearman’s correlation and linear regression analyses confirmed moderate to strong agreement between the two methods. In conclusion, CLIA showed strong concordance with and comparable diagnostic accuracy to ELISA for AIBD serodiagnosis. Furthermore, this is the first study to evaluate CLIA for autoantibody detection in blister fluid from BP patients, demonstrating that blister fluid may serve as a less invasive sample type for diagnosis. Our cut-off analysis in a treated cohort also suggested that manufacturer-recommended thresholds may yield false-negative results, highlighting the need for population-specific reference intervals.

## 1. Introduction

**Figure 1:**
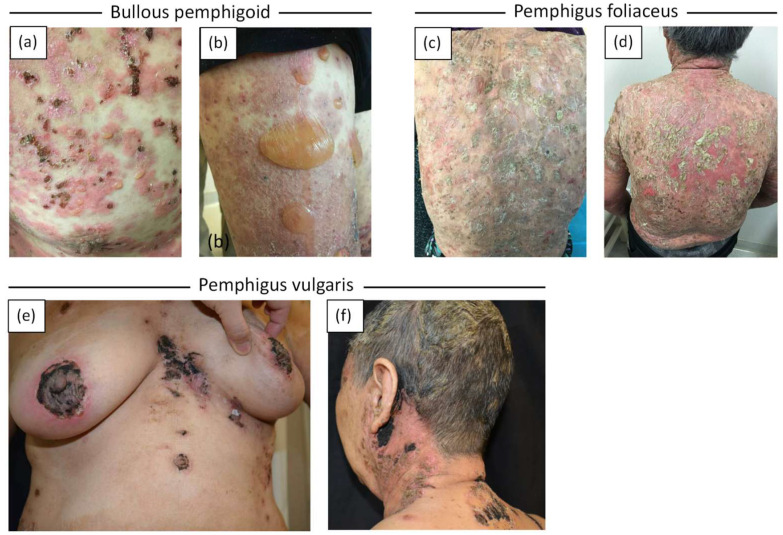
Clinical manifestations of autoimmune bullous diseases. Bullous pemphigoid: Tense blisters arising on erythematous bases, observed on the back (**a**) and arm (**b**) of a patient. Pemphigus foliaceus: Superficial, crusted erosions and erythematous scaly patches on the trunk (**c**) and head (**d**) of a patient. Lesions typically present as “cornflake-like” crusts without mucosal involvement. Pemphigus vulgaris: Flaccid blisters that have ruptured to form widespread, painful erosions with crusts on the chest (**e**) and head (**f**).

Autoimmune bullous diseases (AIBDs), encompassing pemphigus and pemphigoid diseases, are characterized by autoantibodies against skin adhesion proteins, which lead to cutaneous and/or mucosal blistering and erosions [1,2,3,4] (Figure 1). Pemphigus, including pemphigus vulgaris (PV) and pemphigus foliaceus (PF), is driven by autoantibodies against desmogleins (DSG) 1 and 3, structural proteins of desmosomes that maintain intercellular adhesion. The distinct clinical manifestations of pemphigus patients reflect their autoantibody profiles. Nearly all PV patients develop oral mucosal lesions, while those with the mucocutaneous variant also present with skin lesions, typically flaccid blisters. In contrast, PF lesions are confined to the skin and predominantly manifest as erythema [3]. PF involves only anti-DSG1 antibodies, whereas mucosal-dominant PV exclusively involves anti-DSG3, and mucocutaneous PV involves both anti-DSG3 and anti-DSG1 [2,3]. Bullous pemphigoid (BP), the most common pemphigoid subtype, is marked by autoantibodies against BP180 and BP230, components of hemidesmosomes at the dermal–epidermal junction [2,4,5]. BP typically presents with tense blisters and erythema, and mild oral lesions develop in 10–20% of patients [4].

The diagnosis of AIBDs requires the integration of clinical characteristics, histopathology, direct immunofluorescence (IF), and serological detection of circulating autoantibodies [6,7,8,9]. Serological detection and quantification of circulating autoantibodies play an indispensable role in the diagnosis, classification, and monitoring of AIBDs. Specifically, circulating anti-DSG1 and anti-DSG3 antibodies are well-established biomarkers for diagnosis, activity monitoring, and prognosis prediction in pemphigus [10,11,12]. Similarly, anti-BP180 autoantibodies are reliable serological markers for BP [13,14,15,16,17]. Furthermore, combined detection of anti-BP180 and anti-BP230 can enhance diagnostic sensitivity for BP compared with anti-BP180 alone [18]. Anti-BP180 and anti-BP230 are also detectable in blister fluid, and their levels show a strong correlation with corresponding serum concentrations [19]. Therefore, although blister fluid is not yet a routine sample in clinical practice, it serves as a minimally invasive alternative to serum for detecting BP-related antibodies [19].

Enzyme-linked immunosorbent assay (ELISA) is widely used in the serological testing for AIBDs due to its capacity to provide quantitative, antigen-specific antibody measurements in a cost-effective manner [7]. However, ELISA has several limitations, including a relatively narrow detection range and a slow turnover time.

In recent years, chemiluminescent immunoassay (CLIA), an advanced immunoassay platform that utilizes luminescence-based technology, has proven to be a valuable tool for measuring autoantibodies in multiple diseases [20,21,22]. In this study, we employed magnetic particle-based acridinium ester direct chemiluminescence technology on an iFlash system. The CLIA platform offers enhanced operational efficiency through full automation and shortened turnover time, reducing the detection time from approximately 2 h (ELISA) to 20 min (CLIA). Furthermore, CLIA exhibits a broader dynamic range and higher sensitivity in detecting antibodies at low concentrations, improving the diagnostic accuracy for autoimmune diseases [23].

With the widespread availability of commercial CLIA kits, many clinical institutions are transitioning from ELISA to CLIA for the diagnosis of AIBDs. Some patients initially tested by ELISA are later followed up using CLIA. Given that autoantibody levels are critical for disease activity monitoring in AIBDs, both clinicians and patients may question whether the results from the two assays are comparable or directly interchangeable. Previously, Fujio et al. [24] reported that a chemiluminescent enzyme immunoassay (CLEIA) demonstrated comparable reliability to ELISA for monitoring clinical disease activity in pemphigus and pemphigoid. Another study by Zhang et al. [25] also indicated that CLIA showed strong agreement with indirect IF, and provided a wider detection range and greater sensitivity than ELISA. However, existing studies are confined to serum samples. In the present study, we detected anti-BP180, anti-BP230, anti-DSG1, and anti-DSG3 antibodies in serum samples from patients with BP, PV, and PF, as well as from healthy controls (HC). Furthermore, we measured anti-BP180 and anti-BP230 antibodies in blister fluid samples from BP patients and HC. Subsequently, the diagnostic performance of ELISA and CLIA and the correlation between the two assays were evaluated, providing a comprehensive comparison of their clinical applicability. To our knowledge, this is the first study to validate the utility of CLIA in blister fluid samples. Furthermore, our prospectively collected cohort included patients with different treatment statuses and various common comorbidities, which reflects the value and necessary adjustment of commercial kits in real-world clinical settings.

## 2. Results

### 2.1. Demographic and Clinical Characteristics of Serum Donors

A total of 255 serum samples were included in this study, including 163 from patients with BP, PV, or PF, and 92 from HC. The demographic and clinical characteristics of these patients are summarized in Table 1. Among them, 55 BP patients (65.5%), 37 PV patients (53.6%), and 2 PF patients (20%) had active clinical manifestations including blisters, erythema, or mucosal involvement. Furthermore, 71 BP patients (84.5%), 52 PV patients (94.5%), and 9 PF patients (90%) had received therapy in the two months preceding sample collection. The most common comorbidities among the patients were cardiovascular disease (24.5%), diabetes (16.0%), and tumors (12.3%).

### 2.2. Diagnostic Performance of Serum Antibodies with Laboratory Cut-Off Values

The diagnostic performance of serum antibodies was evaluated using established laboratory cut-off values for both ELISA and CLIA (Table S1). Anti-BP180 and anti-BP230 were applied for the diagnosis of BP, anti-DSG1 for PF, and anti-DSG1/3 for PV. To assess the diagnostic accuracy of anti-BP180 and anti-BP230 for BP, sera from PV and PF patients served as disease controls in addition to HC. Conversely, the sera from BP patients were used as disease controls when evaluating the diagnostic accuracy of anti-DSG1 or anti-DSG3 for pemphigus. Both ELISA and CLIA demonstrated acceptable diagnostic performance for anti-BP180, anti-DSG1, and anti-DSG3 in our cohort, with accuracy values approaching or exceeding 0.85. Comparative analysis revealed assay-specific profiles: ELISA exhibited superior clinical sensitivity, while CLIA showed enhanced specificity for anti-BP180 and anti-DSG3 detection.

In the context of BP diagnosis, anti-BP180 outperformed anti-BP230, with a significantly higher accuracy in both ELISA (McNemar test, *p* = 0.002) and CLIA (McNemar test, *p* = 0.001). For anti-BP180, both assays demonstrated perfect specificity, moderate sensitivity, and comparable accuracy (ELISA: 0.878, CLIA: 0.871, McNemar test, *p* = 0.815).

Regarding the optimization of cut-off thresholds for pemphigus diagnosis, the 20 U/mL cut-off provided superior diagnostic performance for both anti-DSG1 and anti-DSG3 in ELISA, achieving significantly higher accuracy (McNemar test, *p* < 0.05). For anti-DSG1, CLIA demonstrated superior accuracy relative to ELISA even when applying the 20 U/mL cut-off (McNemar test, *p* = 0.008). For anti-DSG3, however, CLIA did not show a significant difference from ELISA (cut-off value: 20 U/mL) in the accuracy (McNemar test, *p* = 0.289).

The agreement between ELISA and CLIA, based on established laboratory cut-off values, is presented in Table 2. The two assays demonstrated excellent concordance in measuring serum anti-BP180 antibodies, with a concordance rate of 92.9% (Cohen’s κ = 0.807), while the level of agreement for anti-BP230 was lower (Cohen’s κ = 0.762). For the detection of anti-DSG1 and anti-DSG3, the agreement between the two assays was significantly higher with the ≥20 U/mL cut-off for ELISA, achieving concordance rates exceeding 90% (Cohen’s κ = 0.780 and 0.882 for anti-DSG1 and anti-DSG3, respectively).

### 2.3. ROC Analysis

**Figure 2:**
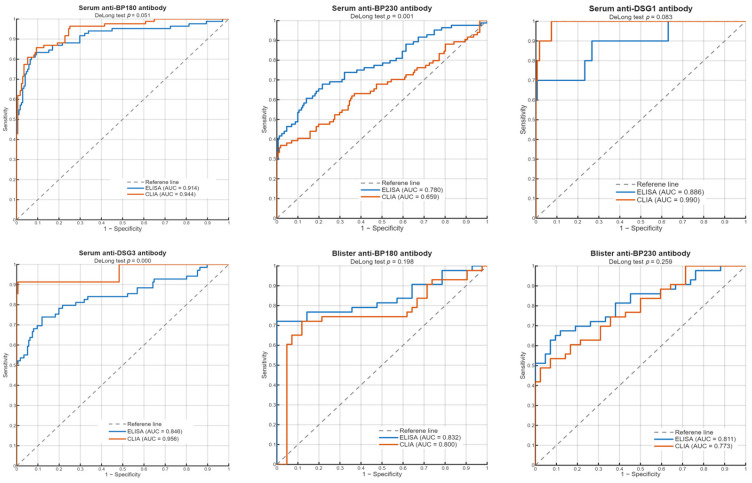
Receiver operating characteristic curve analysis.

ROC analysis was performed to evaluate the diagnostic performance of ELISA and CLIA using both serum and blister fluid samples. HC and disease controls were combined as the control group. As summarized in Table 3 and Figure 2, serum anti-BP180, anti-DSG1, and anti-DSG3 antibodies demonstrated robust diagnostic performance in both assays, with area under the curve (AUC) values exceeding 0.8. Notably, CLIA showed higher AUC values for these three antibodies. In contrast, ELISA outperformed CLIA in detecting anti-BP230, achieving a higher AUC of 0.780. However, statistically significant differences between the two methods were only observed for serum anti-BP230 and anti-DSG3 (DeLong test, *p* < 0.05).

The optimal cut-off values determined by Youden index analysis revealed significant discrepancies from the laboratory-recommended cut-offs for CLIA, where all calculated cut-offs were lower than the standard 20 AU/mL threshold.

ROC analysis of blister fluid revealed that anti-BP180 antibodies showed good diagnostic performance for BP, with AUC values exceeding 0.8 in both methods. Diagnostic performance of anti-BP230 also remained within an acceptable range. ELISA yielded higher AUC values, though the difference was not statistically significant (DeLong test, *p* > 0.1).

### 2.4. Interassay Comparison

#### 2.4.1. Spearman’s Correlation Analysis

Spearman’s correlation analysis (Table 4) revealed good concordance between ELISA and CLIA, though the strength of association varied according to both antibody specificity and sample type. For all antibodies examined, correlations in both serum and blister fluid were positive and statistically significant (*p* < 0.05). In serum samples, antibody-specific correlation coefficients ranged from 0.524 (anti-BP230) to 0.745 (anti-BP180). In contrast, blister fluid analysis demonstrated strong correlations (correlation coefficients > 0.8) for both anti-BP180 and anti-BP230. Notably, anti-BP180 consistently exhibited the highest degree of interassay correlation irrespective of the sample source.

#### 2.4.2. Linear Regression Analysis

**Figure 3:**
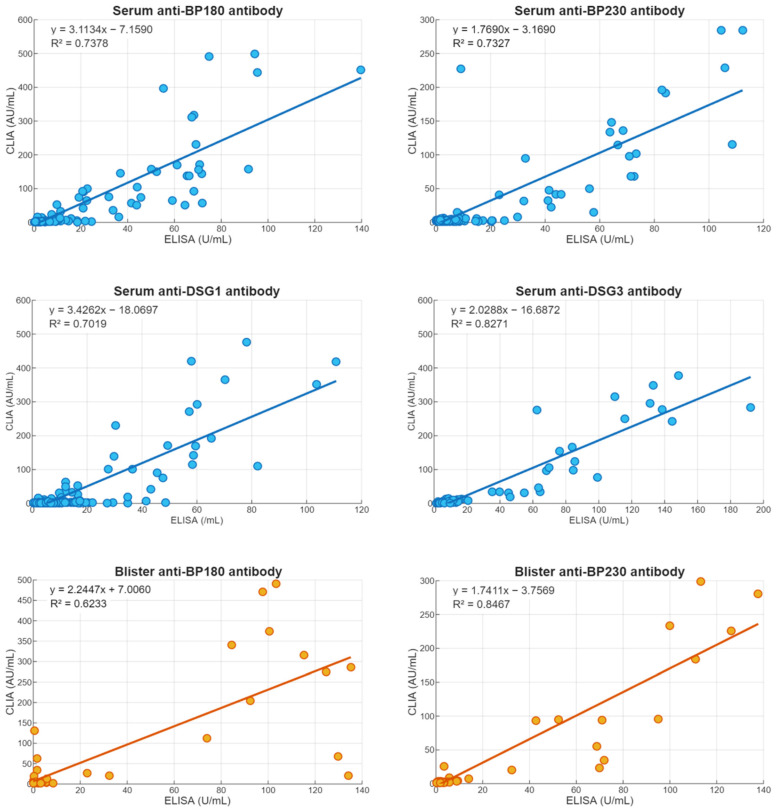
Linear regression analysis.

Linear regression analysis was performed to derive conversion equations for estimating CLIA values from ELISA measurements, using only data points within the quantitative detection range (Figure 3). In serum samples, strong linear correlations were observed between the two platforms, with coefficient of determination (R^2^) values of 0.738 for BP180, 0.733 for BP230, 0.696 for DSG1, and 0.827 for DSG3, indicating that 70-83% of the variability in CLIA values could be explained by ELISA measurements. For blister fluid samples, the R^2^ values for anti-BP180 and anti-BP230 were 0.623 and 0.847, respectively, reflecting moderate to strong linear associations. Importantly, although significant correlations were observed for several antibodies, the substantial inter-individual variability limits the reliable prediction of CLIA values from ELISA measurements.

### 2.5. Association with Clinical Manifestations

Patients were classified into two groups based on the presence or absence of active clinical manifestations, including blisters, erythema, or mucosal involvement (Table 1). Among them, 55 BP patients and 40 pemphigus patients presented with at least one active manifestation, whereas 29 BP and 39 pemphigus patients exhibited no such findings. Serum antibody levels were compared between these groups using the Mann–Whitney U test. Overall, the two assays demonstrated comparable performance in distinguishing patients with different clinical statuses. However, given that CLIA offers a broader detection range, the differences were more pronounced when comparing mean or median antibody concentrations.

In BP patients, neither anti-BP180 nor anti-BP230 levels measured by either assay showed a significant difference between those with and without active manifestations (*p* > 0.05). However, the presence of blisters was associated with significantly higher anti-BP180 levels (ELISA: median 68.3 U/mL vs. 18.4 U/mL, *p* = 0.003; CLIA: median 149.7 AU/mL vs. 36.2 AU/mL, *p* = 0.012). By contrast, anti-BP230 levels were not associated with blister formation.

Pemphigus patients with active manifestations had significantly higher levels of both anti-DSG1 (ELISA: median 41.1 U/mL vs. 5.1 U/mL, *p* < 0.001; CLIA: median 124.8 AU/mL vs. 4.2 AU/mL, *p* = 0.003) and anti-DSG3 (ELISA: median 66.7 U/mL vs. 10.1 U/mL, *p* = 0.005; CLIA: median 115.1 AU/mL vs. 5.5 AU/mL, *p* = 0.009) antibodies compared to those without active manifestations. Furthermore, anti-DSG1 levels were significantly higher in pemphigus patients with cutaneous lesions (blisters or erythema) than in those without (*p* < 0.001), whereas anti-DSG3 levels did not differ significantly between these two groups. Conversely, anti-DSG3 levels were significantly higher in patients with mucosal lesions (*p* < 0.001), while anti-DSG1 levels were not.

## 3. Discussion and Conclusions

This study presents an evaluation of ELISA and CLIA for the detection of four key autoantibodies (anti-BP180, anti-BP230, anti-DSG1, and anti-DSG3) in pemphigus and BP. Our findings confirm that CLIA is a highly reliable and specific method for the serodiagnosis of AIBDs. Furthermore, our study highlights the potential of blister fluid as an alternative sample type in the diagnosis of BP. Although CLIA is not yet included in most AIBD diagnostic guidelines, our findings may support its future adoption.

ELISA has been widely used in the diagnosis of AIBDs, whereas CLIA has gained attention for its high degree of automation, efficiency, and broad dynamic range. Magnetic bead-based CLIA exhibits faster antibody–antigen binding kinetics than plate-based ELISA. This advantage comes from its larger surface area and suspension state, which enable autoantibodies to access surface-bound antigens more efficiently than on the diffusion-limited planar surface of microplates [26,27]. Additionally, magnetic separation in CLIA simplifies the washing process and lowers background signals. Unlike the enzyme-based signal generation system in ELISA, the CLIA platform in our study employs acridinium ester. This compound emits light directly upon triggering, which shortens the reaction time and eliminates the need for enzymatic reactions that are vulnerable to environmental interference (e.g., temperature or pH fluctuations). Photomultiplier-based quantification of amplified chemiluminescent signals in CLIA also provides significantly higher analytical sensitivity than that achieved by spectrophotometric chromogen detection in ELISA [28]. Therefore, CLIA can detect low-concentration autoantibodies with greater accuracy, facilitating early diagnosis of autoimmune diseases. In our real-world study, CLIA generally exhibited higher AUC values than ELISA, although the difference was not statistically significant. However, CLIA still has several limitations, including higher cost and the requirement of specialized equipment.

Previous studies have already evaluated luminescence-based technologies for diagnosing AIBDs, demonstrating that CLIA exhibits diagnostic value comparable to or superior to ELISA [24,25]. Fujio et al. [24] evaluated a newly developed CLIEA method integrated with the STACIA1 system (LSI Medience Corporation, Tokyo, Japan) for serum-based diagnosis of pemphigus and BP. However, the assay did not include the detection of anti-BP230. Another study by Zhang et al. [25], which used the same CLIA kit as in our study, similarly reported that CLIA outperformed ELISA and was comparable to indirect IF for AIBD diagnosis. However, like the study by Fujio et al. [24], they did not include blister fluid analysis. By including blister fluid specimens, our study fills this gap. Notably, blister fluid collection is constrained by inherent anatomical factors. Patients with BP typically present with tense bullae that facilitate fluid aspiration [4,5], while pemphigus patients characteristically exhibit flaccid blisters that easily rupture, making it difficult to collect adequate blister fluid samples [3]. Accordingly, the present study was restricted to blister fluid samples from BP patients.

Our study shows that both ELISA and CLIA demonstrated robust diagnostic performance for BP and achieved acceptable concordance using blister fluid samples. These findings align with previous reports that antibody levels in serum and blister fluid were significantly correlated [29,30,31], suggesting that blister fluid may serve as a viable, less invasive alternative to serum in the diagnosis of AIBDs, particularly BP. However, it must be acknowledged that blister fluid analysis yielded lower area under the curve (AUC) values and wider 95% confidence intervals compared to serum. Therefore, we suggest that blister fluid may serve as a useful supplement to serum. However, it should not be relied upon as a standalone diagnostic tool.

In terms of serum antibodies, both assays demonstrated robust diagnostic performance, though with distinct strengths. ELISA exhibited higher sensitivity for anti-BP180, anti-BP230, and anti-DSG3, while CLIA demonstrated superior specificity for all four antibodies. The relatively lower sensitivity observed with CLIA may be attributed to two main factors. Firstly, certain CLIA platforms may be less effective in detecting autoantibodies that require extended antigen–antibody incubation periods, which may limit antibody detection under standard conditions [32,33]. Secondly, the inclusion of a large proportion of treated patients in our cohort likely reduced circulating autoantibody levels. This latter point may also explain why the optimal diagnostic cut-offs derived from ROC analysis for CLIA were significantly lower than the manufacturer-recommended threshold of 20 AU/mL. These findings underscore the manufacturer’s recommendation for laboratories to establish their own reference intervals based on local population characteristics. Similarly, for pemphigus diagnosis, increasing the ELISA cut-off from 7 or 14 U/mL to 20 U/mL improved specificity and overall accuracy in our analysis.

Spearman’s correlation analysis confirmed a moderate-to-strong positive correlation between ELISA and CLIA for all antibody measurements. Consistent with these findings, linear regression analysis indicated a strong correlation between the methods. However, in accordance with previous reports by Fujio et al. [24], CLEIA and ELISA indices may exhibit substantial discrepancies within the same sample. These discrepancies partly stem from differences in calibration systems and antigen expression systems. The ELISA in our work employs a two-point linear calibration (0 and 100 U/mL), assuming linearity across the entire concentration range. However, given the sigmoidal nature of antigen–antibody binding kinetics, this assumption may cause inaccurate estimation. In contrast, CLIA employs a master curve pre-established by the manufacturer, which is adjusted during each run using four calibrators to generate a final working curve. While this approach improves efficiency and automation, it may introduce bias if the current reagent lot differs from the master curve lot. Furthermore, ELISA uses a baculovirus expression system to generate DSG1 and DSG3, whereas CLIA uses Chinese hamster ovary cells. Notably, the baculovirus expression system does allow post-translational modifications similar to those in mammalian cells [34]. Although the conformational epitopes of DSG1 and DSG3 do not rely on post-translational modifications such as glycosylation [35], slight differences in protein structure, purity, or aggregation state between the two systems can still affect autoantibody–antigen binding efficiency. Additionally, differences in solid-phase geometry and signal generation systems may also contribute to the observed discrepancies. Consequently, despite the correlations identified, quantitative results from CLIA and ELISA are not directly interchangeable at the individual patient level due to considerable inter-individual variability.

Therefore, laboratories are recommended to use a consistent method for the serial monitoring of disease activity, as switching between platforms could lead to misinterpretation of longitudinal changes in antibody titers. In our study, both ELISA and CLIA showed comparable ability to distinguish patients by clinical manifestations based on *p* values. However, due to its wider dynamic range, CLIA revealed more pronounced differences in antibody levels between active and stable patients. This suggests that CLIA may offer better resolution for disease activity monitoring.

**Figure 4:**
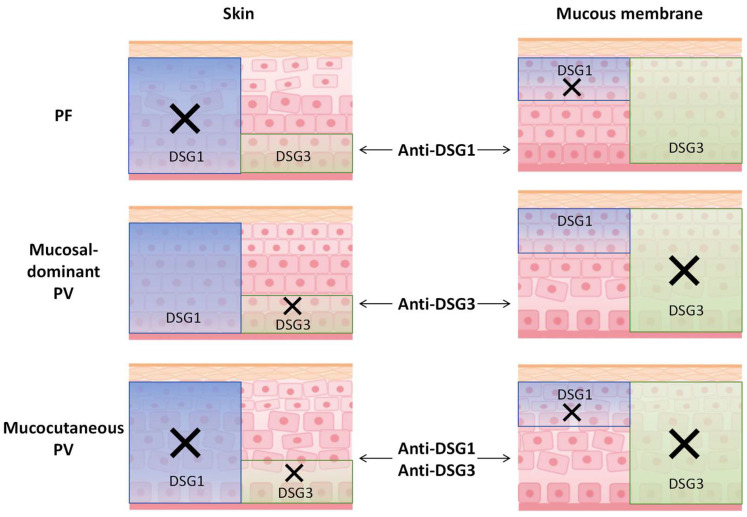
Desmoglein compensation hypothesis. DSG1 is expressed throughout the epidermis but concentrated in the superficial layers of mucous membranes. DSG3 is expressed throughout mucous membranes but concentrated in the basal layers of the epidermis. Either DSG1 or DSG3 alone is sufficient to maintain cell–cell adhesion. In PF patients (anti-DSG1 alone), only superficial skin blisters develop. In mucosal-dominant PV patients (anti-DSG3 alone), only mucous membranes are affected. In mucocutaneous PV patients (both anti-DSG1 and anti-DSG3), both suprabasal skin blisters and mucous membrane lesions occur. Abbreviations: PF, pemphigus foliaceus; PV, pemphigus vulgaris; DSG, desmoglein.

This study further compared the levels of antibodies in patients with different clinical manifestations. In pemphigus patients, disease activity correlated with anti-DSG1 and anti-DSG3 antibody levels, consistent with earlier studies [10,12]. Importantly, anti-DSG1 levels correlated with cutaneous lesions, while anti-DSG3 levels correlated with mucosal lesions, supporting the desmoglein compensation hypothesis [36,37]. This hypothesis is based on the differential expression patterns of DSG1 and DSG3 across tissues, and the idea that either DSG1 or DSG3 alone can sustain cell–cell adhesion (Figure 4). In mucosal tissue, Dsg3 is expressed throughout the entire epithelium, with higher expression in the basal layers, while Dsg1 expression is very low and confined to the superficial layers. In cutaneous epidermal tissue, Dsg1 is expressed throughout the epidermis, with higher expression in the superficial layers, while Dsg3 is concentrated in the basal layer [36]. Consequently, autoantibodies against DSG1 alone primarily cause cutaneous lesions, whereas autoantibodies against DSG3 alone cause mucosal lesions.

For BP, we observed that anti-BP180, but not anti-BP230, levels were significantly linked to the presence of blisters. This finding reinforces the specific pathogenic role of anti-BP180, whose interaction with Fc receptors is critical for blister formation [2], and supports prior evidence identifying anti-BP180 as the key marker for activity monitoring in BP [16,17]. This difference stems from structural distinctions between the two antigens. BP180 is a transmembrane protein whose immunodominant NC16A domain is exposed on the cell surface, allowing anti-BP180 antibodies to trigger complement activation, inflammatory cell recruitment, and basement membrane degradation [1,38]. In contrast, BP230 is an intracellular cytoplasmic protein, which is normally inaccessible to circulating autoantibodies [39]. Consequently, anti-BP230 antibodies are predominantly generated as secondary events through epitope spreading following initial tissue injury [39], which explains their lack of direct correlation with active blister formation.

This study has several limitations. A substantial proportion of patients were on treatment and in a stable disease stage at the time of serum collection, which may have led to an underestimation of the true diagnostic sensitivity of both assays. Moreover, as serum and blister fluid samples were obtained from different patient subsets, a direct intra-individual comparison between the two sample types was not feasible. Future studies incorporating paired samples from larger, longitudinal cohorts would be valuable to validate and extend our findings.

## 4. Materials and Methods

### 4.1. Study Cohort and Sample Collection

This study was conducted in the Department of Dermatology at Peking Union Medical College Hospital and received ethical approval from the Institutional Review Board (K23N0268).

All serum or blister fluid samples from patients were prospectively collected and stored at −80 °C until analysis. Inclusion criteria comprised: (1) age ≥ 18 years, and (2) a confirmed diagnosis of BP, PV, or PF based on typical clinical manifestations and laboratory evidence (including histopathology, direct IF, indirect IF, or indirect IF on salt-split skin). Exclusion criteria included: (1) insufficient clinical data and (2) concurrent immunodeficiencies, malignancies, or active systemic infections.

Control samples consisted of serum from healthy volunteers and blister fluid from patients with non-immune-mediated bullous dermatoses.

A total of 255 serum samples were analyzed, including 92 from HC, 84 from BP, 69 from PV, and 10 from PF. Additionally, 85 blister fluid samples were evaluated, consisting of 42 from controls and 43 from BP patients. Each sample was aliquoted for parallel testing using ELISA and CLIA.

### 4.2. ELISA

Anti-DSG1 and anti-DSG3 antibodies were quantified using the MESACUP-2 DSG1/DSG3 ELISA kits (Medical & Biological Laboratories Co. Ltd., MBL, Nagoya, Japan), while anti-BP180 and anti-BP230 were detected using the MESACUP BP180/BP230 ELISA kit (MBL). Medium-binding 48-well microtiter plates were coated with recombinant antigens: the NC16a domain of BP180 produced as a fusion protein in *Escherichia coli* (*E. coli*), the N- and C-terminal regions of BP230 produced in *E. coli*, and DSG1 and DSG3 produced as secreted proteins using a baculovirus expression system.

According to the manufacturer, all reagents and samples were equilibrated to room temperature (20–30 °C) prior to use. Serum samples were diluted 1:101 with Assay Diluent. Then, 100 µL of calibrators or diluted samples was added to antigen-coated microwells and incubated for 60 min at room temperature. Wells were then washed four times with diluted Wash Solution. Subsequently, 100 µL of conjugated reagent (horseradish peroxidase-labeled goat anti-human IgG) was added to each well and incubated for another 60 min, followed by a second washing step (four times). After that, 100 µL of substrate solution (3,3′,5,5′-tetramethylbenzidine dihydrochloride/hydrogen peroxide) was added and incubated for 30 min. The enzymatic reaction was stopped by adding 100 µL of stop solution (1.0 N sulfuric acid). The microplate was subsequently transferred to a Sunrise microplate absorbance reader (Tecan Austria GmbH, Grödig, Austria) for a single-wavelength read at 450 nm. The optical measurement was conducted utilizing narrow-band interference filters with a bandwidth of 10 ± 2 nm and a wavelength accuracy of ±2 nm, powered by a 20 W halogen lamp light source. Antibody concentrations (U/mL) were determined via linear interpolation based on a two-point calibrator system comprising Calibrator 1 (negative control, 0 U/mL) and Calibrator 2 (positive control, 100 U/mL). All raw optical data were automatically processed, recorded, and saved via the Magellan clinical software (version 4.50, Tecan Austria GmbH).

Laboratory cut-off values defined by the manufacturer’s guidelines were: negative for anti-BP180 and anti-BP230 (<9 U/mL), anti-DSG1 (<14 U/mL), and anti-DSG3 (<7 U/mL); positive for anti-DSG1 and anti-DSG3 (≥20 U/mL). No established cut-off value was available for blister fluid samples.

### 4.3. CLIA

CLIA was performed using commercially available kits on a fully automated 3000-G chemiluminescence immunoassay analyzer (Shenzhen Yhlo Biotech Co. Ltd., Shenzhen, China), operated via the iFlash Operating Software (version 1, Shenzhen Yhlo Biotech Co. Ltd.). The assay employed paramagnetic beads coated with recombinant BP180, BP230, DSG1, or DSG3 antigens. Similarly to ELISA, recombinant BP180 NC16a and BP230 were produced in *E. coli* as fusion proteins. However, DSG1 and DSG3 were produced in Chinese hamster ovary cells.

A 10 µL aliquot of the sample (serum or blister fluid) was incubated with the antigen-coated superparamagnetic microparticles at 37 °C for 10 min. After magnetic washing, acridinium-labeled mouse anti-human IgG was added and incubated for another 10 min at 37 °C. Following a second wash, pre-trigger and trigger solutions were added to initiate the chemiluminescent reaction. The emitted light signal was captured by the system’s internal photomultiplier tube, and the resulting relative light units (RLU) were measured. Antibody concentrations (AU/mL) were calculated using a four-point calibration curve adjusted by a master curve provided via barcode.

The detection ranges for both sample types were established at 1–500 AU/mL for BP180, 1–300 AU/mL for BP230, 1–600 AU/mL for DSG1, and 1–400 AU/mL for DSG3. The laboratory cut-off value for a positive result was defined as ≥20 AU/mL for serum antibodies. Similarly to ELISA, no cut-off value was recommended for blister fluid samples by the manufacturer.

### 4.4. Statistical Analysis

Statistical analysis was performed using MATLAB R2025b (MathWorks, Natick, MA, USA), RStudio (version 4.4.1, Posit Software, PBC, Boston, MA, USA), and Microsoft Office Professional 2024 for Windows (Microsoft Corporation, Redmond, WA, USA), with statistical significance set at *p* < 0.05.

Diagnostic performance of each assay, including sensitivity, specificity, and accuracy, was evaluated using established laboratory cut-off values, and accuracy was compared using McNemar’s test. Agreement between the two methods was assessed using 2 × 2 contingency tables and Cohen’s kappa coefficient. Diagnostic capacity of assays was evaluated through receiver operating characteristic (ROC) curve analysis, complemented by the DeLong test. Quantitative association between the assays was measured by Spearman’s correlation. Linear regression analysis was applied to develop conversion equations for predicting CLIA values based on ELISA measurements. Serum antibody levels in patients with different clinical manifestations were compared by the Mann–Whitney U test.

## Figures and Tables

**Table 1:** Demographic and clinical data of serum donors.

	BP	PV	PF
Participants	84	69	10
Age, mean ± SD (year)	70.6 ± 12.7	55.7 ± 12.3	60.5 ± 7.4
Sex			
Male	54	31	4
Female	30	38	6
Clinical manifestation			
Blister	41	9	1
Erythema	47	22	3
Mucosal lesion	7	27	1
Therapy within 2 months			
Yes	71	66	9
None	13	3	1
Underlying disease			
Cardiovascular disease	27	11	2
Diabetes	17	7	2
Tumor	17	3	0
None	28	38	0

Abbreviations: BP, bullous pemphigoid; PV, pemphigus vulgaris; PF, pemphigus foliaceus; SD, standard deviation.

**Table 2:** Concordance rate between ELISA and CLIA for serum antibody detection.

			CLIA (AU/mL)		Concordance Rate			Kappa
			Negative (<20)	Positive (≥20)	All	Positive	Negative	
ELISA (U/mL)	Anti-BP180	Negative (<9)	185	1	0.929	0.852	0.953	0.807
		Positive (≥9)	17	52				
	Anti-BP230	Negative (<9)	211	0	0.941	0.795	0.966	0.762
		Positive (≥9)	15	29				
	Anti-DSG1	Negative (<14)	186	4	0.871	0.686	0.919	0.610
		Positive (≥14)	29	36				
	Anti-DSG1	Negative (<20)	207	7	0.941	0.814	0.965	0.780
		Positive (≥20)	8	33				
	Anti-DSG3	Negative (<7)	167	0	0.796	0.581	0.865	0.476
		Positive (≥7)	52	36				
	Anti-DSG3	Negative (<20)	211	0	0.969	0.900	0.981	0.882
		Positive (≥20)	8	36				

Abbreviations: ELISA, enzyme-linked immunosorbent assay; CLIA, chemiluminescence immunoassay.

**Table 3:** Receiver operating characteristic curve analysis.

Sample	Method	Antibody	AUC (95% CI)	Youden Index Analysis			
				Optimal Cut-Off	Sensitivity	Specificity	Youden Index
Serum	ELISA	BP180	0.914 (0.870–0.958)	6.8	0.810	0.930	0.740
		BP230	0.780 (0.715–0.845)	4.8	0.607	0.860	0.467
		DSG1	0.886 (0.762–1.000)	44.6	0.700	0.994	0.694
		DSG3	0.846 (0.782–0.910)	8.2	0.739	0.881	0.620
	CLIA	BP180	0.944 (0.915–0.973)	3.1	0.857	0.906	0.763
		BP230	0.658 (0.579–0.737)	7.3	0.369	0.982	0.351
		DSG1	0.990 (0.975–1.000)	3.4	1.000	0.926	0.926
		DSG3	0.956 (0.917–0.995)	1.0	0.913	0.994	0.907
Blister	ELISA	BP180	0.832 (0.738–0.926)	15.7	0.721	1.000	0.721
		BP230	0.811 (0.719–0.904)	3.0	0.651	0.905	0.556
	CLIA	BP180	0.800 (0.698–0.903)	19.5	0.721	0.925	0.646
		BP230	0.773 (0.674–0.873)	3.0	0.535	0.929	0.464

Abbreviations: AUC, area under the curve; CI, confidence interval; ELISA, enzyme-linked immunosorbent assay; CLIA, chemiluminescence immunoassay.

**Table 4:** Spearman’s correlation analysis between ELISA and CLIA.

Sample	Antibody	Spearman’s Correlation Coefficient (95% CI)	*p*
Serum	Anti-BP180	0.745 (0.662–0.814)	<0.001
	Anti-BP230	0.524 (0.413–0.630)	<0.001
	Anti-DSG1	0.617 (0.514–0.698)	<0.001
	Anti-DSG3	0.650 (0.566–0.728)	<0.001
Blister	Anti-BP180	0.865 (0.783–0.912)	<0.001
	Anti-BP230	0.825 (0.694–0.906)	<0.001

Abbreviations: ELISA, enzyme-linked immunosorbent assay; CLIA, chemiluminescence immunoassay; CI, confidence interval.

## Data Availability

The original data are available from the corresponding author on reasonable request.

## Supplementary Materials

The following supporting information can be downloaded at: https://www.mdpi.com/article/10.3390/ijms27146272/s1.
