# Supplementary material for: Chemiluminescence Immunoassay and Enzyme-Linked Immunosorbent Assay in the Diagnosis of Pemphigoid and Pemphigus: A Comparative Study
Source: Int J Mol Sci. 2026 Jul 14;27(14):6272. doi: 10.3390/ijms27146272 (PMC13411565; doi:10.3390/ijms27146272)
Supplement: Supplementary file 1 [file ijms-27-06272-s001.zip › ijms-4255940-supplementary.pdf]

Table S1. Diagnostic performance of serum antibodies with laboratory cut-off values.

Method	Antibody	Cut-off	Group HC (*n* = 92)	BP (*n* = 84)	PV (*n* = 69)	PF (*n* = 10)	Sensitivity	Specificity	Accuracy
ELISA (U/ml)	BP180	Negative (<9)	86	23	67	10	0.726	0.953	0.878
		Positive (≥9)	6	61	2	0			
	BP230	Negative (<9)	88	47	66	10	0.440	0.959	0.788
		Positive (≥9)	4	37	3	0			
	DSG1	Negative (<14)	76	73	38	3	0.700	0.847	0.839
		Positive (≥14)	16	11	31	7			
	DSG1	Negative (<20)	89	79	43	3	0.700	0.955	0.941
		Positive (≥20)	3	5	26	7			
	DSG3	Negative (<7)	73	69	17	8	0.754	0.807	0.792
		Positive (≥7)	19	15	52	2			
	DSG3	Negative (<20)	87	83	31	10	0.551	0.966	0.849
		Positive (≥20)	5	1	38	0			
CLIA (AU/ml)	BP180	Negative (<20)	92	32	68	10	0.619	0.994	0.871
		Positive (≥20)	0	52	1	0			
	BP230	Negative (<20)	91	56	69	10	0.333	0.994	0.776
		Positive (≥20)	1	28	0	0			
	DSG1	Negative (<20)	92	84	36	3	0.700	1.000	0.984
		Positive (≥20)	0	0	33	7			
	DSG3	Negative (<20)	92	84	33	10	0.522	1.000	0.865
		Positive (≥20)	0	0	36	0			

Abbreviations: HC, healthy control; BP, bullous pemphigoid; PV, pemphigus vulgaris; PF, pemphigus foliaceus; ELISA, enzyme-linked immunosorbent assay; CLIA, chemiluminescence immunoassay
